# Supplementary material for: Mutations in PCYT2 disrupt etherlipid biosynthesis and cause a complex hereditary spastic paraplegia
Source: Brain. 2019 Oct 22;142(11):3382–97. doi: 10.1093/brain/awz291 (PMC6821184; doi:10.1093/brain/awz291)
Supplement: awz291_Supplementary_Data [file awz291_supplementary_data.zip › awz291-Suppl_data/Supplementary_Data4.zip!/Supplementary information 4 - plasma lipidomics.zip!/Plasma lipidomics/Control vs Patient2/Statistical plots and data/Random forest.pdf]

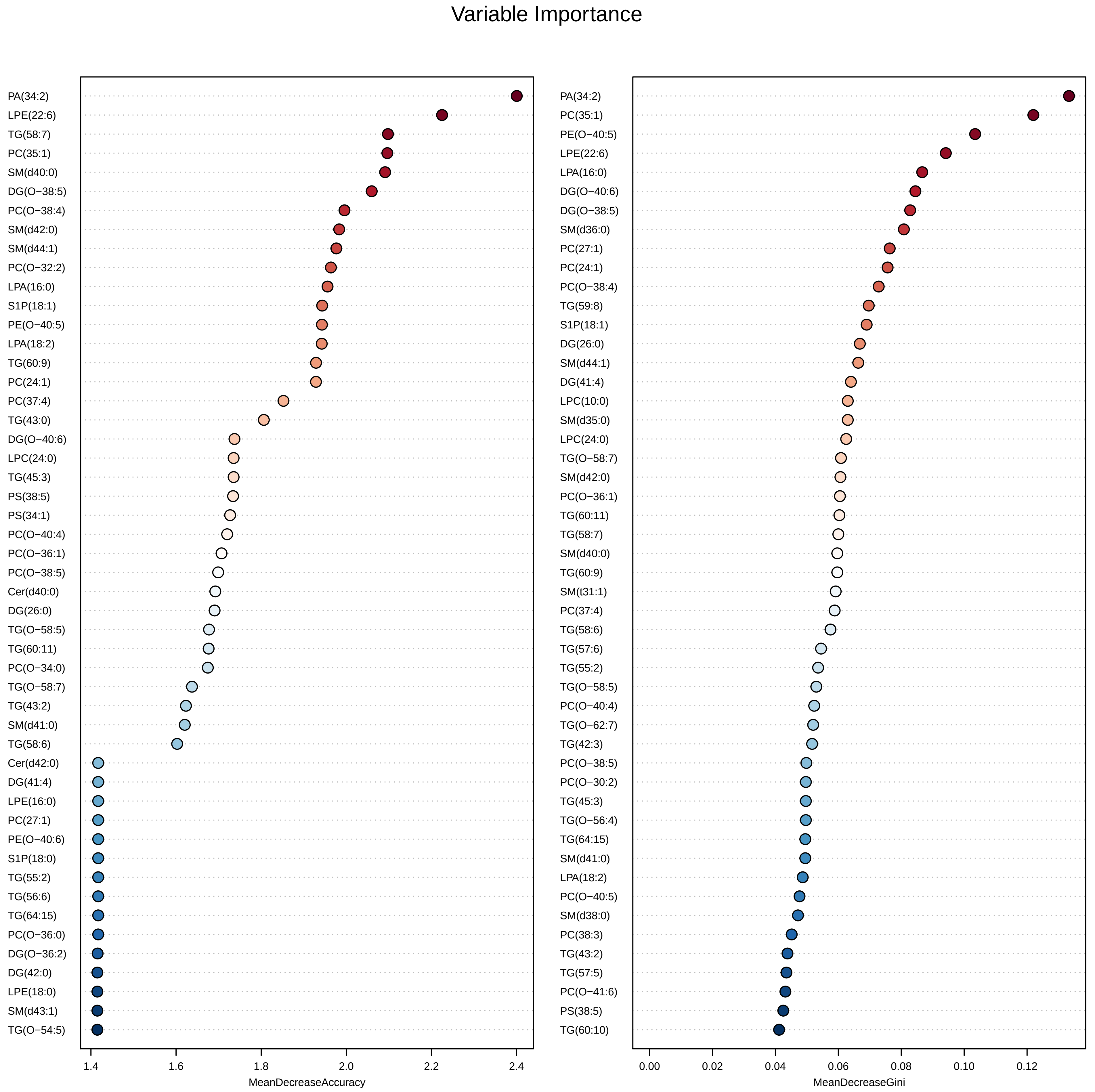

Variable Importance
PA(34:2)
LPE(22:6)
TG(58:7)
PC(35:1)
SM(d40:0)
DG(O−38:5)
PC(O−38:4)
SM(d42:0)
SM(d44:1)
PC(O−32:2)
LPA(16:0)
S1P(18:1)
PE(O−40:5)
LPA(18:2)
TG(60:9)
PC(24:1)
PC(37:4)
TG(43:0)
DG(O−40:6)
LPC(24:0)
TG(45:3)
PS(38:5)
PS(34:1)
PC(O−40:4)
PC(O−36:1)
PC(O−38:5)
Cer(d40:0)
DG(26:0)
TG(O−58:5)
TG(60:11)
PC(O−34:0)
TG(O−58:7)
TG(43:2)
SM(d41:0)
TG(58:6)
Cer(d42:0)
DG(41:4)
LPE(16:0)
PC(27:1)
PE(O−40:6)
S1P(18:0)
TG(55:2)
TG(56:6)
TG(64:15)
PC(O−36:0)
DG(O−36:2)
DG(42:0)
LPE(18:0)
SM(d43:1)
TG(O−54:5)
1.4
1.6
1.8
2.0
2.2
2.4
MeanDecreaseAccuracy
PA(34:2)
PC(35:1)
PE(O−40:5)
LPE(22:6)
LPA(16:0)
DG(O−40:6)
DG(O−38:5)
SM(d36:0)
PC(27:1)
PC(24:1)
PC(O−38:4)
TG(59:8)
S1P(18:1)
DG(26:0)
SM(d44:1)
DG(41:4)
LPC(10:0)
SM(d35:0)
LPC(24:0)
TG(O−58:7)
SM(d42:0)
PC(O−36:1)
TG(60:11)
TG(58:7)
SM(d40:0)
TG(60:9)
SM(t31:1)
PC(37:4)
TG(58:6)
TG(57:6)
TG(55:2)
TG(O−58:5)
PC(O−40:4)
TG(O−62:7)
TG(42:3)
PC(O−38:5)
PC(O−30:2)
TG(45:3)
TG(O−56:4)
TG(64:15)
SM(d41:0)
LPA(18:2)
PC(O−40:5)
SM(d38:0)
PC(38:3)
TG(43:2)
TG(57:5)
PC(O−41:6)
PS(38:5)
TG(60:10)
0.00
0.02
0.04
0.06
0.08
0.10
0.12
MeanDecreaseGini
